# Evaluating the Accuracy of Virtual Reality Trackers for Computing Spatiotemporal Gait Parameters

**DOI:** 10.3390/s21103325

**Published:** 2021-05-11

**Authors:** Michelangelo Guaitolini, Fitsum E. Petros, Antonio Prado, Angelo M. Sabatini, Sunil K. Agrawal

**Affiliations:** 1 The BioRobotics Institute, Scuola Superiore Sant’Anna, 56127 Pisa, Italy; angelo.sabatini@santannapisa.it; 2 Department of Excellence in Robotics & AI, Scuola Superiore Sant’Anna, 56127 Pisa, Italy; 3 Department of Rehabilitative and Regenerative Medicine, Columbia University Medical Center, New York, NY 10032, USA; jap2254@columbia.edu (A.P.); sa3077@columbia.edu (S.K.A.); 4 Department of Mechanical Engineering, School of Engineering and Applied Science, Columbia University, New York, NY 10027, USA

**Keywords:** virtual reality, gait analysis, gait event detection, gait features, motion analysis

## Abstract

Ageing, disease, and injuries result in movement defects that affect daily life. Gait analysis is a vital tool for understanding and evaluating these movement dysfunctions. In recent years, the use of virtual reality (VR) to observe motion and offer augmented clinical care has increased. Although VR-based methodologies have shown benefits in improving gait functions, their validity against more traditional methods (e.g., cameras or instrumented walkways) is yet to be established. In this work, we propose a procedure aimed at testing the accuracy and viability of a VIVE Virtual Reality system for gait analysis. Seven young healthy subjects were asked to walk along an instrumented walkway while wearing VR trackers. Heel strike (HS) and toe off (TO) events were assessed using the VIVE system and the instrumented walkway, along with stride length (SL), stride time (ST), stride width (SW), stride velocity (SV), and stance/swing percentage (STC, SWC%). Results from the VR were compared with the instrumented walkway in terms of detection offset for time events and root mean square error (RMSE) for gait features. An absolute offset between VR- and walkway-based data of (15.3 ± 12.8) ms for HS, (17.6 ± 14.8) ms for TOs and an RMSE of 2.6 cm for SW, 2.0 cm for SL, 17.4 ms for ST, 2.2 m/s for SV, and 2.1% for stance and swing percentage were obtained. Our findings show VR-based systems can accurately monitor gait while also offering new perspectives for VR augmented analysis.

## 1. Introduction

Gait-related movement analysis is a complex discipline involving various factors. It has various applications in research, such as quantitatively describing gait as well as diagnosing and evaluating of the subject’s locomotion abilities. Gait is an indicator of overall health as it dictates autonomy. Disease and accidental injuries are common factors for abnormal gait. Moreover, motor-related pathologies become more frequent with aging, affecting 10% of people between 60 and 70 years old and 60% of those over the age of 80 [1]. In this challenging scenario, gait analysis has become an area of interest for many research groups both for clinical application and quantitative assessment.

Currently, there are several methods to observe and extract important gait characteristics. The state-of-the-art in this field relies on camera-based systems, more specifically stereophotogrammetry systems. These systems may depend on infra-red cameras and reflective markers or on marker-less video recording and shape recognition [2,3,4]: the former method is the most accurate while being burdensome in terms of subject’s preparation, while the latter is more agile in terms of preparation, although it is less accurate.

These systems are able to reconstruct body position with millimetric accuracy, thus precisely evaluating movements and gait features [5]. However, their cost and need for specific indoor instrumented environments present practical limitations to their applications. 

More versatile, low-cost systems are represented by wearable sensors such as inertial motion units (IMUs) [6] or instrumented clothing [7]. Wearable sensors allow motion analysis in various environments. Although, wearables are able to track and extract gait characteristics [8], they require calibration procedures to re-align sensor outputs. Furthermore, technology-related issues, such as sensor drift or magnetic disturbances, present a significant impediment for long-term applications. Calibration procedures often need a-priori knowledge of the task being examined [9]. Additionally, the output from these sensors, mainly acceleration and angular velocity, need to be integrated to get position information, which introduces reconstruction error into the analysis [10]. Several methods have been proposed to tackle these issues [9,11,12], however, IMUs still result in inconsistent and less accurate systems when compared to camera-based setups.

Instrumented walkways represent a non-wearable solution for gait analysis and require shorter set-up time as compared to camera-based systems. These walkways rely on pressure sensors placed within a flexible mat that may be used to define a path and perform gait trials. The walkways detect gait events based on footfall pressures on the mat surface and compute gait parameters from the corresponding foot position information from the walkway [13]. The walkway does not require the user to wear any particular instrumentation, so the user’s state is not altered.

Instrumented walkways are widely used in gait analysis [14,15,16] and found their application in rehabilitation procedures when spatiotemporal parameters were needed. Instrumented walkways have been demonstrated to be a useful tool when 3D motion analysis is not needed; they also do not need any dress-up/marker placement procedure that is typically needed for camera-based systems. However, the walkway’s potential is limited by its size and shape, which usually only allows for straight-line gait tracking, as sidesteps and turnarounds may not be fully captured. 

Although, the standard instrumented walkways can accurately measure gait features, they are expensive. Moreover, performing gait analysis in a condition close to everyday life would capture a more realistic information. Therefore, a portable and affordable solution for gait analysis could have a significant impact on motion studies and diagnostic procedures.

Recently, the use of virtual reality (VR) in rehabilitation and motion analysis has increased. Clinicians use it as an innovative method to collect movement data while augmenting conventional clinical care and optimizing the physical abilities of their patients [17,18]. VR may be defined as a simulation of a real-world environment, generated through computer software [18]. The VR environment is experienced by the user through a human-machine interface [19]. Previous research highlights the capability of VR systems to enhance skills acquisition and retention by providing task specificity (ecological validity), repetition and external real-time feedback (knowledge of results) [20]. 

VR systems use two laser emitter units, called Lighthouses, for position tracking [21]. These emitters alternate horizontally and vertically to scan the environment in each direction. VR headsets, trackers and controllers are equipped with photodiodes that are marked during Lighthouse scans. The difference in time when the various photodiodes are hit by the laser allows position and orientation reconstruction. Furthermore, some VR systems, such a VIVE systems, integrate this method with IMU-based tracking which allows for higher update rates [21].

For several clinical populations, VR based gait training with feedback was beneficial in improving parameters such as gait symmetry and walking speed [22]. The usage of VR systems both in combination with other sensors and by themselves has been tested [23] and showed promising results. For this reason, research is moving in that direction.

For instance, Peruzzi et al [24] examined the effect of VR-based gait training on multiple sclerosis patients. They observed how patients significantly improved their walking endurance and speed, as well as, cadence, stride length, lower limb range of motion, and power. Furthermore, patients also experienced an improvement in balance. Similarly, Wang et al [25] investigated how VR training may improve balance in patients with Parkinson’s disease (PD). Researchers reviewed studies involving VR effects on PD patients - a total of 12 studies with a median PEDro score of 6.4 involving 419 participants. The review reported significant improvements in Berg Balance Scale, Time Up and Go Test (TUG), and stride length in PD patients who received VR-based treatment as compared to patients treated using traditional procedures.

Despite their reported advantages, VR systems still represent a relatively new technology that need to be validated for gait analysis. Recently, VR systems’ tracking was validated against camera-based systems. The comparison showed that 3D tracking was accurate within (0.7 ± 0.3) cm translationally and (1.64 ± 0.18)° rotationally. This results suggest that VR sensors can be used to accurately track joint motion for clinical and research applications, although accuracy on extracted parameters was not discussed [26]. 

Although validated against camera-based systems, VR’s potential for accurately detecting clinically relevant and reliable information has yet to be investigated. A cheaper solution with respect to cameras for spatiotemporal parameters assessment is represented by IMU-based setups. IMU-based setups may show good accuracy in tracking human motions, however, specific sensor fusion algorithms are usually needed to achieve that performance [6]. Although, VR systems are spatially limited (they must be used indoors and calibrated), they do provide a more consistent and reliable position tracking since they, unlike IMUs, directly provide displacement information [26]. Furthermore, VR trackers can be used for long-term data acquisition without the added complexity for incorporating additional trackers [27]. Given its accuracy against well-known systems, VR has the potential to be a reliable, low-cost gait analysis system representing a valid alternative to wearable setups for indoor environments. Table 1 summarizes the main features of various motion capture systems, from camera-based to wearables and instrumented walkways. It presents a perspective about current state-of-the-art in motion analysis to refer to when testing VR system.

In this paper, we propose an estimation and calculation procedure to validate a VIVE virtual reality system against an instrumented walkway, here used as a reference. The VR system consisted of a headset and trackers. The method uses event detection to segment the data and estimate the spatiotemporal during overground walking. To the best of our knowledge, the VIVE system has not been methodically characterized as a gait analysis tool. Furthermore, we will make the tools to record the VIVE tracker data and calculate the gait parameters with a range of shoe sizes publicly available. The tools will be available for download in a GitHub repository and we will include the link will with the final version of this paper.

## 2. Materials and Methods

Seven healthy young adults participated in the experiments (one female, six males, 25 ± 5 years old). Subjects were informed about the research procedure and signed a written consent form before participating. All participants had a normal or corrected-to-normal vision; none of the participants had any reported disorders and all were new to the experimental conditions.

### 2.1. Procedure

**Figure 1:**
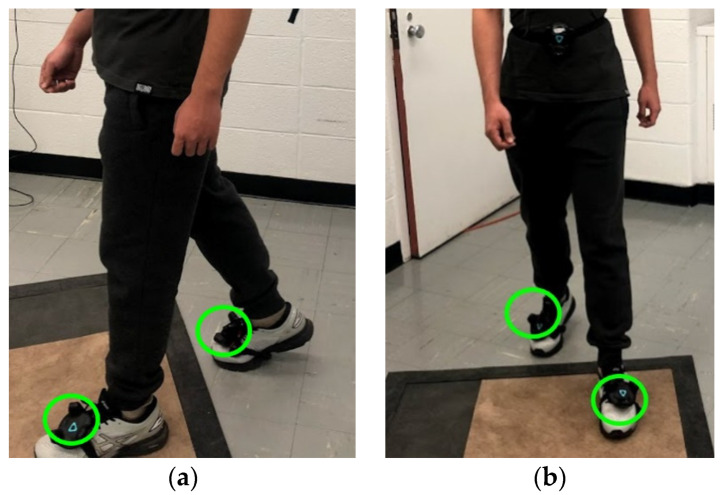
VIVE sensors placed on participants’ foot upper back similar to reference [28]. (**a**,**b**) The VIVE trackers are circled in green. The sensors were secured with an attachment tied to the shoes and oriented to face in the direction of the toes for consistency in measurement.

Participants walked on a 6.10 m × 0.61 m instrumented walkway (Zeno Walkway, Protokinetics, Havertown, PA, USA) at their own speed for twenty laps, back-and-forth, while wearing VR trackers on their feet (Figure 1). Trackers were placed on front feet, on the upper back of the subject’s shoes. Sensor placement on shoes allows for accurate tracking of feet motion [28].

**Figure 2:**
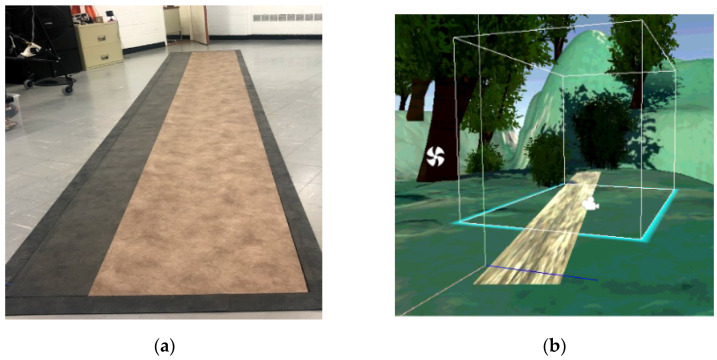
(**a**) Physical walkway setup in the laboratory (**b**) and corresponding walkway configuration in the virtual environment.

A Unity3D (Unity Technologies, San Francisco, CA, USA) program was developed to display a virtual walkway within the VR headset (VIVE HTC PRO, Valve Corporation, Bellevue, WA, USA) paired with STEAM VR v020 [29]. The virtual environment (VE) was calibrated so that the virtual walkway was aligned and mapped one-to-one with respect to the physical walkway. The visual environment was a 3D outdoor space (Figure 2); objects such as trees and animals were placed to provide depth reference. Participants experienced the virtual environment and motions in it in the first-person perspective, while their body was not rendered in the virtual environment. The lack of self-image may influence gait analysis as it was demonstrated by previous studies [30]. However, this condition is consistent for any trial under exam, since we did not investigate physical-world gait trials.

The session trial consisted of twenty consecutive laps on the walkway. Participants were asked to walk straight without stepping outside of the walkway and to perform turnarounds outside the walkway so that they could be easily excluded from data analysis. VR tracker trajectories in the VR were measured at a sampling frequency of 90 Hz while the walkway registered events at 120 Hz. The data was concurrently recorded using a custom inbuilt user datagram protocol (UDP) packet sent at the start of the walkway recording session and the walkway data were downsampled to 90 Hz for analysis.

Data recorded by the instrumented walkway and computed with the accompanying software: ProtoKinetics Movement Analysis Software (PKMAS 5.09C3, ProtoKinetics, Havertown, PA, USA), were used as ground truth values to test VR system performances in gait analysis.

**Figure 3:**
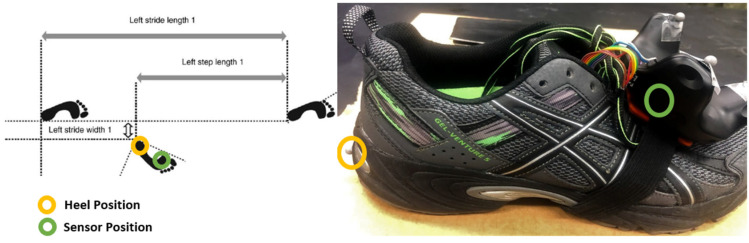
Schematic SL and SW from walkway [31]: position of the VR tracker on the foot upper back (green) and heel position (yellow) used for said features calculations.

Finally, it should be noted that gait features are usually computed from heel position, as seen in Figure 3, as provided in the manual for the walkway, while VR sensors are placed on the feet between toe and ankle Figure 3. Therefore, a body axis transformation was carried out to shift the current position of the tracker to the heel for a more appropriate comparison. The 3D distance between the heel and the foot sensors were taken using a camera-based motion tracking system (Vicon, Oxford, UK). The heel position was determined using the position and rotation of the sensors and then applying the transformation. A reference table containing sensor to heel 3D displacement for several shoe sizes is included in the appendix (Appendix A).

The camera-based system was used to take static measures on trackers’ position on shoes. As mentioned earlier, we decided to rely on the instrumented walkway for the VR system validation because it represents a portable, accurate tool for gait analysis. 

### 2.2. Event Detection

#### 2.2.1. VR Event Detection

MATLAB R2020a (MathWorks, Inc., Natick, MA, USA) was used for off-line signal processing. VR trackers trajectories were filtered using a third-order forward-backward low-pass Butterworth filter with a cutoff frequency of 12 Hz [32]; trajectories were also detrended using a linear detrend function.

The function used to gather information about the different gait phases is performed using feet trackers trajectories. The segmentation procedure divides the gait cycle after detecting two main events: toe-off (TO) and heel strike (HS).

**Figure 4:**
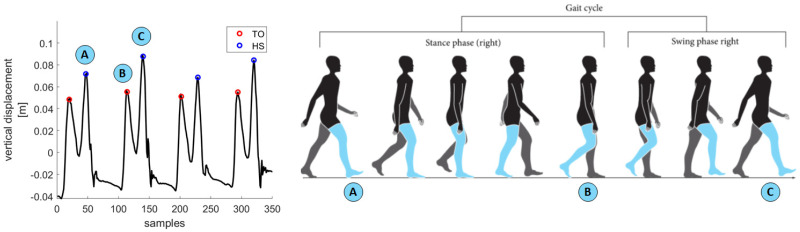
Four consecutive gait cycles and their corresponding HS (blue) and TO (red) detected from vertical displacement of a tracker on the foot. Vertical displacement is shown on the *y*-axis while the *x*-axis reports samples over time of the signal. A schematic representation of a gait cycle is also reported to clearly indicate which events correspond to the peaks on the signal. Negative vertical displacement depends on VR 3D space calibration: the 0 level may not correspond with ground level and could result in negative vertical values for the feet trackers.

The analysis is performed while the subject is already in motion so as to avoid transitory phases. In order to do so, the subject was asked to take a couple of strides before walking on the walkway. The tracker trajectory can be described by a double-peaked waveform (Figure 4). The first peak corresponds to the foot lifting from the ground, i.e. TO, as specified in [33], and it determines a transition from stance to swing phase. The second peak, namely the foot reaching the ground again, corresponds to a HS. TO and HS are also depicted in Figure 4 as red and blue dots, respectively. After correctly detecting HS and TO, it is possible to segment each cycle in swing (SW) and stance (ST) phases.

Time intervals between TO and subsequent HS are swing phases (SWC), and intervals between HS and TO are stance phases (STC) of a gait cycle. The sum of swing and corresponding stance determines the whole gait cycle.

The vertical trajectory from a foot tracker is shown in Figure 4 and the associated cyclical waveform was analyzed to detect events. The algorithm proposed in this paper represents a simple and straightforward method to detect gait events. The method is based on those available for camera-based data [33] as trajectories in a global reference frame were available for VR systems similar to the camera-based systems. In the research described in [33] both the heel and toe trajectories were available, while we only had mid-foot trajectories. So, reported waveforms and proposed algorithms may result differently, even if they were based on the same principles.

#### 2.2.2. Walkway Event Detection

PKMAS dedicated software was used for gait event detection on walkway data and signal processing. Gait events were defined using the procedure described in the manual for the instrumented walkway [34]. Heel strikes (HS) are defined as first contact instants, they correspond to the initial time (frame) that the foot comes in contact with the walkway. Toe offs (TO) are defined as last contact instants and correspond to the last time (frame) that the foot is in contact with the walkway.

For this purpose, the foot is represented by its bidimensional footfall. The footfall is computed as the ellipse with the smallest area that completely encloses all of the activated sensors of a footprint, computed with the minimum area bounding ellipse algorithm. Footprint recognition and gait event evaluation are performed as detailed in [34].

### 2.3. Gait Features

#### 2.3.1. VR Gait Features Evaluation

Gait events detected using VR data were used to evaluate gait features (Figure 3): stride length (SL), stride time (ST), stride width (SW), stride velocity (SV), stance percentage (STC%) as well as swing percentage (SWC%) were evaluated. These parameters were chosen as they represent some of the most commonly evaluated features in gait analysis. As such, it was of our interest to test the VR system ability in computing them. Furthermore, other parameters such as joint angles, would be available only by adding more VR trackers. Gait phases were reported as percentage of the whole gait cycle since it is the most common way to report them in gait analysis. Features were computed in 2D since the walkway can only provide 2D displacement information. Each feature was computed as follows: 

Stride length of the i-th stride is computed as:

(1) $$SL_{i}=\sqrt{{\left(FTT_{X}^{HS\left(i\right)}-FTT_{X}^{HS\left(i-1\right)}\right)}^{2}+{\left(FTT_{Y}^{HS\left(i\right)}-FTT_{Y}^{HS\left(i-1\right)}\right)}^{2}}$$

where $FTT_{X}^{HS\left(i\right)}$ and $FTT_{X}^{HS\left(i-1\right)}$ are the foot tracker position at the i-th and (i−1)-th HS events respectively. X and Y are the anteroposterior and the mediolateral components of the position vectors respectively; the anteroposterior axis is pointing forward while the mediolateral axis is pointing to the left.

Stride time of the i-th stride:

(2) $$ST_{i}=t_{HS\left(i\right)}-t_{HS\left(i-1\right)}$$

where $t_{HS\left(i\right)}$ and $t_{HS\left(i-1\right)}$ are the time instants corresponding to the i-th and (i−1)-th HS events. 

Stride width is computed as the perpendicular distance between the line made by two consecutive same-foot heel strikes and the contralateral heel, as specified in the walkaway manual [34]. The stride width is then computed as follows using the position of the feet at heel strike and mediated over each cycle, as shown in Figure 3:

(3) $$SW_{i}=\sqrt{{\left(RFT_{X}^{HS\left(i\right)}-LFT_{X}^{HS\left(i-1\right)}\right)}^{2}+{\left(RFT_{Y}^{HS\left(i\right)}-LFT_{Y}^{HS\left(i-1\right)}\right)}^{2}-{\left(\frac{SL_{i}}{2}\right)}^{2}}$$

(4) $$\mathrm{Stride}\;\mathrm{velocity}\;\mathrm{is}\;\mathrm{computed}\;\mathrm{as}\;\mathrm{stride}\;\mathrm{length}\;\mathrm{over}\;\mathrm{stride}\;\mathrm{time}\;\mathrm{ratio}:\;SV=\frac{SL}{ST}$$

Stance and swing percentages are computed as the portion of stance phase and swing phase over the whole gait cycle, expressed as percentages. Given that they are competing percentages, an offset recorded for STC% will represent a negative offset for the SWC%:

(5) $$STC\%=100\times \frac{stance\_duration\left[sec\right]}{cycle\_duration\left[sec\right]}$$

(6) $$SWC\%=100\times \frac{swing\_duration\left[sec\right]}{cycle\_duration\left[sec\right]}$$

It should be noted that STC%+SWC%=100.

#### 2.3.2. Walkway Gait Features Evaluation

The walkway uses the gait events detected, specifically heel strike, to evaluate gait features: meaning, the stride length (SL), stride time (ST), stride width (SW), stride velocity (SV), stance percentage (ST%) as well as swing/stance percentage (SWC%, STC%) were all evaluated at heel strike.

### 2.4. Validation Procedure

VR and walkway data were synchronized using a custom algorithm (UDP packet) during experimental sessions. Following offline data processing, VR-based feature validation was performed. A comparison between gait events (HS and TO) was carried out by determining the time difference between the two systems. The sensitivity, precision and accuracy are reported.

The sensitivity is the percentage of gait events that are detected successfully by the VR system, within 33.3 ms resolution (3 timestamps for 90 Hz). This timestamp was chosen to represent a significant sensitivity as well as a reliable resolution for accurately describing a gait cycle. Timestamps were chosen based on the reliability testing as well as a review of the algorithm results presented in [35]. Outlier values were identified as values higher than 3.5 times the median absolute deviation (MAD) around the median [36]. A total of 3.39% for HS (40 events out of 1185) and 2.2% for TO (27 events out of 1227) were identified as outliers. These outlier values were a result of sensor disconnection and subsequent loss of tracking. Outliers were removed at the event identification and were not used for the spatiotemporal calculations:

(7) $$Sensitivity=100\times \frac{TP}{TP+FN}$$

True positives (TP) are the values from the walkway that were detected by the VR, while false negatives (FN) are events that were identified by the walkway but undetected by the VR system. Accuracy and precision are described by the time offset error and its distribution [37].

The closest HS and TO temporal matches within a 33.3 ms resolution were used to determine possible differences between the VR system and the ground truth, namely the walkway. The mean error, absolute mean error, standard deviation as well as the root mean square error (RMSE) are presented to show the magnitude and spread of differences in measurements between the VR system and the reference walkway.

## 3. Results

A detailed analysis of temporal and spatiotemporal parameters from the proposed VR setup and the walkway reference are presented below.

### 3.1. Validation of Gait Events

**Figure 5:**
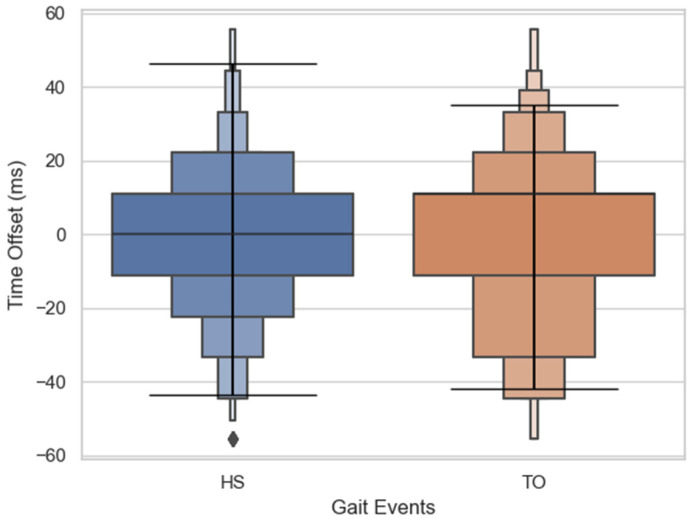
Detection time offset for HS and TO gait events by the VR as compared to the walkway is presented above, with the horizontal line indicating where ~95% of the errors fall (mean ± 1.96 × SD). VR is able to detect a significant portion of the events within ±33.3 ms.

Our system was able to identify the gait events (heel strikes and the toe offs) that were detected by the walkway. Figure 5 below shows the detection time offsets between the VR system and the reference walkway. The results show that about 90% of the gait events are detected within a 33.3 ms window. Mean absolute error, standard deviation, and sensitivity are reported in Table 2, while Figure 5 below shows that the proposed method was able to detect all HSs within a window of 15.3 ± 12.8 ms and TOs within 17.6 ± 14.8 ms.

### 3.2. Validation of Gait Features

**Figure 6:**
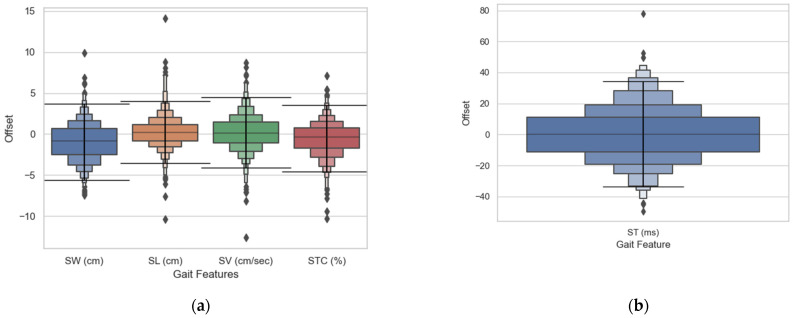
VR offset compared to matched events measured from the walkway. Where the horizontal line delimits where ~95% of the errors fall (mean ± 1.9 × SD).

**Figure 7:**
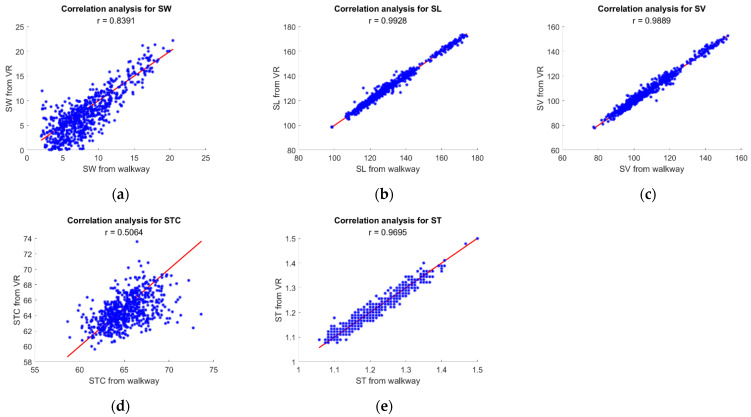
Correlation plots between the VR- and the walkway-based gait features. Red lines show a one-to-one relation and the dots indicate where the values fall. The correlation coefficients are reported in figures.

A detailed comparison of gait features is given below (Table 3). On average, the mean absolute error of all the subjects shows that there is 2.0 cm and 1.4 cm difference between the spatial parameters (SW and SL respectively), 17.4ms offset for the stride time, and about 1.6% difference between the stance-swing percentage. Figure 6 and Figure 7 show the error distribution along with the ~95% limitations and the correlation of the VR vs. walkway measurements respectively. This analysis was carried out using a 33.3 ms resolution to avoid possible discrepancies from gait event detection offsets.

## 4. Discussion

This study aims to evaluate the viability and accuracy of a VIVE Virtual Reality system for gait characterization; an instrumented walkway was used as a reference against the VR system.

The rationale behind the comparison with a walkway is that this sensor already represents a portable, accurate alternative to camera-based systems. On the other hand, a correctly validated VR system may have a potential that a walkway does not offer, given that VR systems are less expensive, portable and can detect 3D movements. Since VR systems directly provide displacement information in the global reference frame about worn trackers, they are hypothesized to perform more accurately than other wearable technologies such as IMU-based setups against the walkway.

When compared to a more traditional low-cost system for evaluating spatiotemporal parameter, such as IMUs, the VR system showed better accuracy, both in terms of RMS error and mean absolute offset since typical displacement errors with wearable technology are around 5 cm [38,39,40]. This was predictable, since VR systems directly gives displacement information, avoiding sensor-related errors that are typical with IMUs.

Furthermore, gait events were correctly detected with a timing resolution of 33.3 ms and a sensitivity of 88.3% and 94.2% for TO and HS respectively, while corresponding gait features showed a root mean squared error of ~2.3 cm offset for spatial features and ~17 ms of temporal parameters.

### 4.1. Gait Events Detection

Detection of gait events showed high accuracy and resolution, with a mean absolute offset between the two systems of 13.4 ms for the HS and 13.7 ms for the TOs. Event detection discrepancies between the walkway and the VR system may be related to the differences in detection methods and sensors specifications. The walkway dedicated algorithm relied on actual first contacts (heel contacts, mostly) while having the VR trackers placed on the mid-foot needed for different methodologies. Nevertheless, the presented method resulted in line with the walkway and camera-based systems in terms of reliability in gait events detection [38,39,40]. 

Even if only straight-line waking was tested, VR systems allow for observing motions in more complex 3D-spaces, while the reference walkway does not allow that. However, further research is needed to test VR accuracy in assessing spatiotemporal parameters during ambulation in free open space.

Other low-cost wearables, such as IMUs, may be less or more accurate depending on gait speed [41]. Moreover, adding more IMUs to any setup increases the computational load representing a strong limit in terms of sensor network design. VR systems allow for computing gait events using tracker trajectories, as it is usually done with markers and camera-based systems [33]. 

TO detection has lower accuracy as compared to HS. Since TO detection is performed after HS detection, detection errors may accumulate resulting in higher values for TO. Furthermore, as earlier studies point out, the TO is a less drastic movement compared to HS and thus less discernible to detect [38,42]. 

### 4.2. Gait Features Evaluation

The gait feature analysis demonstrates accurate results as compared to the walkway reference for most of the parameters and sensor-position sensitive results for stride width. 

SW and SL showed a mean absolute error of 1.7 cm (3.0 cm and 1.8 cm for 75th percentile and 0.7 cm and 0.5 cm for the 25th percentile, respectively). The distribution of the errors as well as a visual representation of the agreement of the data is presented in Figure 6 and Figure 7 above. These results indicate a better performance compared to other low-cost systems such as IMUs which usually show an error around 3–5 cm [38]. Although camera-based systems or walkways (our reference) show more accuracy, presenting errors of mm, these systems are also far more expensive. 

The SW shows a higher standard deviation and RMSE as compared to SL. This could be attributed to the fact that three sensor positions are needed to compute SW, accumulating possible positional and angular errors from the VR system [26]. The transformation from the foot instep to the heel may result in inaccurate measurements if the sensor is not properly secured. Sensor misplacement and resulting heel position error have a higher effect on SW. Further, because SW is sensitive to heel position conditions where sensors might re-set after a disconnection or get mislabeled might have affected it. Although the reported error shows that the VR system can compete with current wearable systems that report spatiotemporal parameters [40], the error reported is a large percentage compared to the reference walkway (about absolute mean percentage of 30%). Therefore, analysis involving base of support or mediolateral displacement using this method would need alternative methods to get more accurate estimates.

ST showed a mean absolute error of 13.4 ms (19.1 ms for 75th percentile; 5.2 ms for the 25th percentile) which is expected due to the accuracy demonstrated in gait events detection. Similarly, SV showed a mean absolute error of 1.6 cm/s (2.3 cm/s for the 75th percentile; 0.6 cm/s for the 25th percentile). 

The Swing and Stance percentage showed a mean absolute error of 1.6% (2.2% for the 75th percentile; 0.58% for the 25th percentile), as seen in Figure 6 and Figure 7. The STC errors indicate a combined effect of both the stride time error as well as the stance time error, as the STC is the ratio of these two values (Equation (5)). However, similar to previous gait features, these errors are lower than those obtained from other low-cost systems since IMU-based systems showed errors of 30 ± 10 ms [43].

### 4.3. Limits

The procedure for testing the accuracy of VR systems in gait analysis does not consider various speeds (namely, controlled slow walking or running): only straight-line gait at self-selected speed was tested. Moreover, a comparison with a camera-based system could have allowed for testing more versatile scenarios, while the walkway was limited to straight-line walking. Testing VR accuracy during different gait speeds, gait with turns or gait on particular ground would provide a better description of its reliability. Further investigations would provide useful information, although they are beyond the aims of the current study.

Also, it may be noted that the gait events detection algorithm was tailored for healthy subjects only, since our analysis involved only young, healthy participants. The algorithm may not work with certain categories of patients and in that case it could be useful to adopt different gait detection strategies, as described in [44].

Further, this methodology warrants careful placement and securing of the sensors, as misalignment and slipping could impact the sensor-to-heel transformation and as a result stride width computation. 

Despite its low-cost and portability, a VR system is mainly intended for indoor motion tracking providing accuracy at low-cost but only in structured environments. Moreover, the subject would eventually need to wear a VR headset to properly move in the virtual scenario: wearing a VR headset, especially for long sessions, may result in side-effects such as nausea and may result in discomfort for some users [45]. Finally, it should be noted how VR itself may affect user’s motion behaviors.

A test assessing reproducibility over subject and time (namely a test-retest procedure) would have been necessary to have a better perspective on the VR system performances. However, the whole experiment was performed over the course of several weeks for each participant, introducing a variability that may be taken as a proof of reproducibility. 

Despite these limitations, the demonstrated benefits of virtual reality in gait analysis and gait training have been proven [46,47,48] that it holds a great potential for gait analysis.

## 5. Conclusions

We have presented an investigation of the VR system for gait analysis. Our approach consisted of testing how accurately a VR-based setup can detect gait events such as heel strikes and toe offs as well as gait features such as stride length, stride time, stride width, stride velocity and stance/swing percentage. 

This study is intended to offer a direct reference for VR reliability, specifically for determining spatiotemporal parameters during gait. Gait analysis, is typical in the rehabilitation procedure and this study may provide a perspective on a new promising technology, even if more research on pathological gait needs to be performed. Future research will focus on the limitations that affected the proposed approach; namely the VR accuracy at various speeds, its performance with different tasks during walking (e.g., turning or stair climbing) as well as testing VR accuracy against another camera-based system.

We believe that the use of VR-based systems may offer a significant improvement for motion analysis applications. It represents an accurate and low-cost solution that is easily adaptable to any indoor environment while also widening the potential for new testing opportunities to increase the effectiveness of therapeutic treatments.

## Figures and Tables

**Table 1:** Table detailing the state-of-the art for gait analysis and corresponding characteristics.

	Camera-Based Systems	IMUs	Walkways	VR
Collection Space	Dedicated lab	Any environment	Flat surfaces	Any indoor space
Setup time	10 to 20 min, depending on markers setup. Plus, initial configuration.	Few minutes for calibration and sensor placement	Few minutes for initial configuration.	Few minutes for initial configuration
User preparation	Several minutes to sensors markings and identification	Few minutes for sensor placement	No preparation	Few minutes for sensor placement
# of sensors/markers to track a single segment	3	1	-	1
Portability	No	Wearable	Portable	Portable
Cost	Very expensive	Affordable	Expensive	Affordable
Type of measurement	Infrared-based	Inertial and magnetic	Pressure-based	Laser-based
Gait features accuracy (feet displacement)	~0.2 mm	~50 mm	~1 mm	To be investigated
Post-processing time	Up to one hour, depending on marker setup	Minutes	Minutes	Minutes
Covered Gait Features	Spatiotemporal features; 3D-displacement; joint kinematics	Spatiotemporal parameters; joint kinematics	Spatiotemporal features; 2D displacement	Spatiotemporal features; 3D-displacement; joint displacement

**Table 2:** Table showing percentage of HS and TO that can be properly detected in under either 33.3-ms cutoff.

Gait Events	Heel Strike	Toe Off
Cutoff Time Window [ms]	33.3	33.3
Mean Offset [ms]	−2.6 ± 16.9	4.2 ± 17.1
Mean Absolute Offset [ms]	13.4 ± 10.5	13.7 ± 11.0
Sensitivity [%]	94.2	88.3

**Table 3:** Mean offset of VR gait features from matched events (33.3 ms, resolution).

	SW [cm]	SL [cm]	ST [ms]	SV [cm/sec]	Stance-Swing [%]
Mean Offset	−1.0 ± 2.4	0.3 ± 1.9	0.4 ± 17.4	0.2 ± 2.2	0.5 ± 2.1
Mean Absolute Error	2.0 ± 1.6	1.4 ± 1.4	13.4 ± 11.1	1.6 ± 1.5	1.6 ± 1.4
RMS Error	2.6	2.0	17.4	2.2	2.1

## Data Availability

The data that support the findings of this study are available from the corresponding authors, M.G. and F.E.P., upon reasonable request. Also, the functions used for data analysis are available in https://github.com/antoniopradom/VR-Gait-Analysis (accessed on 11 May 2021).

## Appendix A

3D distance from VR tracker to heel (right and left side) for a range of shoe-sizes.

X: in the direction of the length of the foot (shoe) pointing to the heel,

Y: to the right of the foot (shoe), Z: up in the direction of the height of the foot.

**Table A1:** Appendix A.

	US 6		US 9		US 10		US 12	
Shoe Side	L	R	L	R	L	R	L	R
X [mm]	211.04	214.13	225.16	226.76	218.76	241.97	243.43	240.32
Y [mm]	4.21	12.31	14.31	−11.10	3.00	−12.94	7.69	14.64
Z [mm]	−85.98	−86.63	−99.76	−94.29	−91.85	−99.65	98.95	−88.98

## References

[1] Pirker W., Katzenschlager R. (2017). Gait disorders in adults and the elderly: A clinical guide. Wien. Klin. Wochenschr..

[2] Ceseracciu E., Sawacha Z., Cobelli C. (2014). Comparison of markerless and marker-based motion capture technologies through simultaneous data collection during gait: Proof of concept. PLoS ONE.

[3] Della Croce U., Leardini A., Chiari L., Cappozzo A. (2005). Human movement analysis using stereophotogrammetry Part 4: Assessment of anatomical landmark misplacement and its effects on joint kinematics. Gait Posture.

[4] Sandau M., Koblauch H., Moeslund T.B., Aanæs H., Alkjær T., Simonsen E.B. (2014). Markerless motion capture can provide reliable 3D gait kinematics in the sagittal and frontal plane. Med. Eng. Phys..

[5] Merriaux P., Dupuis Y., Boutteau R., Vasseur P., Savatier X. (2017). A study of vicon system positioning performance. Sensors.

[6] Caldas R., Mundt M., Potthast W., Buarque de Lima Neto F., Markert B. (2017). A systematic review of gait analysis methods based on inertial sensors and adaptive algorithms. Gait Posture.

[7] Chen D., Cai Y., Qian X., Ansari R., Xu W., Chu K.C., Huang M.C. (2019). Bring Gait Lab to Everyday Life: Gait Analysis in Terms of Activities of Daily Living. IEEE Internet Things J..

[8] Panero E., Digo E., Agostini V., Gastaldi L. Comparison of Different Motion Capture Setups for Gait Analysis: Validation of spatio-temporal parameters estimation. Proceedings of the MeMeA 2018—2018 IEEE International Symposium on Medical Measurements and Applications.

[9] Latt W.T., Veluvolu K.C., Ang W.T. (2011). Drift-free position estimation of periodic or quasi-periodic motion using inertial sensors. Sensors.

[10] Thong Y.K., Woolfson M.S., Crowe J.A., Hayes-Gill B.R., Jones D.A. (2004). Numerical double integration of acceleration measurements in noise. Meas. J. Int. Meas. Confed..

[11] Liu T., Inoue Y., Shibata K. (2009). Development of a wearable sensor system for quantitative gait analysis. Meas. J. Int. Meas. Confed..

[12] Martin Schepers H., van Asseldonk E.H.F., Baten C.T.M., Veltink P.H. (2010). Ambulatory estimation of foot placement during walking using inertial sensors. J. Biomech..

[13] Lynall R.C., Zukowski L.A., Plummer P., Mihalik J.P. (2017). Reliability and validity of the protokinetics movement analysis software in measuring center of pressure during walking. Gait Posture.

[14] Titianova E.B., Pitkänen K., Pääkkönen A., Sivenius J., Tarkka I.M. (2003). Gait characteristics and functional ambulation profile in patients with chronic unilateral stroke. Am. J. Phys. Med. Rehabil..

[15] Samotus O., Parrent A., Jog M. (2018). Spinal Cord Stimulation Therapy for Gait Dysfunction in Advanced Parkinson’s Disease Patients. Mov. Disord..

[16] Chien S.L., Lin S.Z., Liang C.C., Soong Y.S., Lin S.H., Hsin Y.L., Lee C.W., Chen S.Y. (2006). The efficacy of quantitative gait analysis by the GAITRite system in evaluation of parkinsonian bradykinesia. Park. Relat. Disord..

[17] Sveistrup H. (2004). Motor rehabilitation using virtual reality. J. Neuroeng. Rehabil..

[18] Adamovich S.V., Fluet G.G., Tunik E., Merians A.S. (2009). Sensorimotor training in virtual reality: A review. NeuroRehabilitation.

[19] Burdea G.C. (2003). Virtual Rehabilitation—Benefits and Challenges. Methods Inf. Med..

[20] Cano Porras D., Siemonsma P., Inzelberg R., Zeilig G., Plotnik M. (2018). Advantages of virtual reality in the rehabilitation of balance and gait: Systematic review. Neurology.

[21] Niehorster D.C., Li L., Lappe M. (2017). The accuracy and precision of position and orientation tracking in the HTC vive virtual reality system for scientific research. Iperception.

[22] Lewek M.D., Feasel J., Wentz E., Brooks F.P., Whitton M.C. (2012). Use of visual and proprioceptive feedback to improve gait speed and spatiotemporal symmetry following chronic stroke: A case series. Phys. Ther..

[23] Howard M.C. (2017). A meta-analysis and systematic literature review of virtual reality rehabilitation programs. Comput. Hum. Behav..

[24] Peruzzi A., Zarbo I.R., Cereatti A., Della Croce U., Mirelman A. (2017). An innovative training program based on virtual reality and treadmill: Effects on gait of persons with multiple sclerosis. Disabil. Rehabil..

[25] Wang B., Shen M., Wang Y.X., He Z.W., Chi S.Q., Yang Z.H. (2019). Effect of virtual reality on balance and gait ability in patients with Parkinson’s disease: A systematic review and meta-analysis. Clin. Rehabil..

[26] van der Veen S.M., Bordeleau M., Pidcoe P.E., France C.R., Thomas J.S. (2019). Agreement analysis between vive and vicon systems to monitor lumbar postural changes. Sensors.

[27] Borges M., Symington A., Coltin B., Smith T., Ventura R. HTC Vive: Analysis and Accuracy Improvement. Proceedings of the IEEE International Conference on Intelligent Robots and Systems.

[28] Granqvist A., Takala T., Hämäläinen P., Takatalo J. Exaggeration of avatar flexibility in virtual reality. Proceedings of the CHI PLAY 2018—Proceedings of the 2018 Annual Symposium on Computer-Human Interaction in Play.

[29] Singh Y., Prado A., Martelli D., Petros F.E., Ai X., Mukherjee S., Lalwani A.K., Vashista V., Agrawal S.K. (2020). Dual-Motor-Task of Catching and Throwing a Ball during Overground Walking in Virtual Reality. IEEE Trans. Neural Syst. Rehabil. Eng..

[30] Canessa A., Casu P., Solari F., Chessa M. Comparing real walking in immersive virtual reality and in physical world using gait analysis. Proceedings of the VISIGRAPP 2019—Proceedings of the 14th International Joint Conference on Computer Vision, Imaging and Computer Graphics Theory and Applications.

[31] Huxham F., Gong J., Baker R., Morris M., Iansek R. (2006). Defining spatial parameters for non-linear walking. Gait Posture.

[32] Smith L., Preece S., Mason D., Bramah C. (2015). A comparison of kinematic algorithms to estimate gait events during overground running. Gait Posture.

[33] Rueterbories J., Spaich E.G., Larsen B., Andersen O.K. (2010). Methods for gait event detection and analysis in ambulatory systems. Med. Eng. Phys..

[34] Blvd J.W. ProtoKinetics Movement Analysis Software Measurements and Definitions. https://www.protokinetics.com/pkmas/.

[35] Bruening D.A., Ridge S.T. (2014). Automated event detection algorithms in pathological gait. Gait Posture.

[36] Leys C., Ley C., Klein O., Bernard P., Licata L. (2013). Detecting outliers: Do not use standard deviation around the mean, use absolute deviation around the median. J. Exp. Soc. Psychol..

[37] Linden A. (2006). Measuring diagnostic and predictive accuracy in disease management: An introduction to receiver operating characteristic (ROC) analysis. J. Eval. Clin. Pract..

[38] Storm F.A., Buckley C.J., Mazzà C. (2016). Gait event detection in laboratory and real life settings: Accuracy of ankle and waist sensor based methods. Gait Posture.

[39] Hori K., Mao Y., Ono Y., Ora H., Hirobe Y., Sawada H., Inaba A., Orimo S., Miyake Y. (2020). Inertial Measurement Unit-Based Estimation of Foot Trajectory for Clinical Gait Analysis. Front. Physiol..

[40] Kitagawa N., Ogihara N. (2016). Estimation of foot trajectory during human walking by a wearable inertial measurement unit mounted to the foot. Gait Posture.

[41] Mo S., Chow D.H.K. (2018). Accuracy of three methods in gait event detection during overground running. Gait Posture.

[42] Trojaniello D., Cereatti A., Pelosin E., Avanzino L., Mirelman A., Hausdorff J.M., Croce U. (2014). Della Estimation of step-by-step spatio-temporal parameters of normal and impaired gait using shank-mounted magneto-inertial sensors: Application to elderly, hemiparetic, parkinsonian and choreic gait. J. Neuroeng. Rehabil..

[43] Figueiredo J., Félix P., Costa L., Moreno J.C., Santos C.P. (2018). Gait Event Detection in Controlled and Real-Life Situations: Repeated Measures from Healthy Subjects. IEEE Trans. Neural Syst. Rehabil. Eng..

[44] Zeni J.A., Richards J.G., Higginson J.S. (2008). Two simple methods for determining gait events during treadmill and overground walking using kinematic data. Gait Posture.

[45] Sharples S., Cobb S., Moody A., Wilson J.R. (2008). Virtual reality induced symptoms and effects (VRISE): Comparison of head mounted display (HMD), desktop and projection display systems. Displays.

[46] Ghai S., Ghai I. (2019). Virtual Reality Enhances Gait in Cerebral Palsy: A Training Dose-Response Meta-Analysis. Front. Neurol..

[47] Mirelman A., Patritti B.L., Bonato P., Deutsch J.E. (2010). Effects of virtual reality training on gait biomechanics of individuals post-stroke. Gait Posture.

[48] Moreira M.C., De Amorim Lima A.M., Ferraz K.M., Benedetti Rodrigues M.A. (2013). Use of virtual reality in gait recovery among post stroke patients-a systematic literature review. Disabil. Rehabil. Assist. Technol..

